# Editorial and Miscellaneous

**Published:** 1862-01

**Authors:** 


### EDITORIAL.

Particular Notice.—Until otherwise notified, correspondents are requested to address all communications on business matters of the Journal to

### DR. E. INGALS,

**BOX 14, CHICAGO, ILL.**

Dr. Ingals has kindly consented to act as business manager of the Journal, an arrangement which cannot fail to meet the approbation of all interested, as conducive to the material prosperity of this periodical, and also enabling the editors to devote their attention solely to the duty of appropriately supplying its pages.

Communications for publication may be addressed as heretofore to either of the editors.

☞ Please forward without further delay your arrearages and subscription for 1862, to

**DR. E. INGALS, Box 14, Chicago, Ill.**

---

*Which was it?*—It is variously rumored that a certain personage of high military rank has been invalided for a month or more past, either from typhoid fever, simple catarrhal fever titillated by "attenuations," or a "big scare." *Who is it?*—That is seeking to foist homœopathists upon the medical staff of the army?—That has just secured the position of Brigade Surgeon to a homœopathic confectioner from this city? Will the honorable gentlemen composing the Examining Board at Washington assume the responsibility of this gross insult to the profession, or is it chargeable upon the little gentleman who has thus far been conducting the war upon homœopathic principles—the less done the greater the effect? Or is it a part of the programme by which the valorous * * * hopes to achieve a renomination after having at least once before challenged the forbearance of his constituents? Meanwhile what will the profession do? Are they disposed to submit quietly to being reduced to a level with homœopathists and quacks of every hue? What say the military surgeons, the medical societies and the journals? Have honorable medical men no rights the powers that be are bound to respect? It is somewhat trying to the nerves, with such cases as this in view, to read the spirited rejoinder of our esteemed contemporary, the *Boston Medical and Surgical Journal*, to the *British American Medical Journal*, which groaned in spirit because it had been reported that the notorious "Dr." Tumblety had received the appointment of Staff Surgeon to Gen. McClellan. Our Boston *confrère* could not think this possible, for "they are all honorable men"(!). Whether Mr. Tumblety is Staff Surgeon to Gen. McClellan (son of the late Prof. Geo. B. McClellan of Philadelphia—*quantum mutatus ab illo!*) or not, we do not know, but he may well be, for Chicago can boast a Brigade Surgeon, late a Prof. in a quasi infinitesimal college, confectioner extraordinary to *Small*, and a fitting congener to Tumblety *et id omne genus*.

---

*Early Vaccination.*—Barthez in a memoir read before the Societe Medicale des Hopitaux de Paris concludes that vaccination can scarcely be performed too soon. Yet there is little practical inconvenience in delaying it to the third month as an invasion of small pox before the sixth month is exceedingly rare. He prefers the third month. The *Am. Med. Times* suggests that in the vaccinations rendered necessary by hospital exposures during the first few days after birth, accidents more or less serious and even fatal have sometimes occurred. The three months rule is practically the best.

---

Cæsarean Operation.—Professor Gogefroy describes a successful case of Cæsarean operation lately performed by him. He operated four times, and this is his first successful case. He attributes the recovery to the early period of the operation. The advantages of operating early—if possible, before the rupture of the membranes—are great. The incision into the uterus is, in such a case, much diminished by the contraction of the uterus; in this case, for example, it contracted from about ten to five *centimeters.* Besides injury to the womb and bladder, the consequence of prolonged labor is thereby prevented. The practice, the professor adds, of three nations illustrates this point. The Germans, who operate early, save many females; the French, who delay, save fewer patients; and the English, who only operate *in extremis*, lose almost all their patients.—*British Med. Journal.*

---

Gun-shot Wounds produced during the loading of Artillery.—Dr. Cortese relates (*Omodei Annali Univ. di Med.*) five cases, and gives the following summary of his observation. No other blow of a projectile imparts so great an amount of commotion to the entire limb, and the surgeon is therefore compelled to direct his attention to the whole extremity, whatever amount of lesion may be manifest in the hand. A neglect in this regard may lead to gangrene gaining possession of a large portion of the limb, or to a generalized suppuration, while a diminished power of reaction in the injured parts may give rise to purulent infection, or render amputation useless. When the hand is severely torn, its disarticulation and even the amputation of the forearm is insufficient to secure recovery, because the tissues are more or less destroyed in their intimate structure in consequence of concussion. In such cases, the arm should be amputated. The sooner amputation is performed, the greater is the probability of a favorable result. The rapid and very extensive tumefaction of the limb constitutes a sufficiently certain criterion of the severity of the derangements which are propagated along its whole extent. When no fractures are detected in the diaphysis of the bone, some lesion in the ulnar articulation must be suspected. When the lesion does not seem severe enough to call at once for amputation, we must be prepared for secondary occurrences which will unfit the limb for its functions. Still, conservative treatment should in such cases be attempted.—*British and Foreign Medico-Chirurgical Review.*

---

*Plaster of Paris Dressings for Fractures.*—The Parisian surgeons make frequent use of plaster of Paris dressing for fractures, the method consisting essentially of soaking

4*

layers of coarse cloths in the fluid plaster, applying them lengthwise upon the limb and then encircling all with a common roller bandage, which may or may not be also soaked in the plaster. The whole "sets" quickly, applies itself accurately to the surface and supports equally all the parts. It can readily be so applied that one or more of the splints can be removed at pleasure for the application of lotions, &c. It is unirritating and requires little trouble in adaptation. It is better not to conuect the whole dressing in a mass until the primary swelling has been measurably reduced. The cloth used should be coarse and loose, so as to absorb freely the fluid plaster, which latter should be little thicker than milk or cream.

It is observable that the most of the improvements in surgical practice of the present time consist in simplifications and the discarding of the cumbrous paraphernalia so long in use. And the practice of medicine is likewise freeing itself from divers unnecessary appendages.

---

*Proposed Changes in the Med. Dept. of the Army.*—The bill introduced into the Senate initiates the following arrangement of the medical staff:

Director-General, with rank of Brigadier-General, who shall be chief of the medical corps, aud perform the present Surgeon-General's duties.

Sanitary Inspector-General, with rank of Colonel of Cavaly, who, under the Director-General, shall have general supervision of all that pertains to the sanitary condition of the army.

Six Sanitary Inspectors, with the rank of Lieutenant-Colonel of cavalry, who shall inspect the sanitary condition of the troops, and report to the Sanitary Inspector-General.

Surgeons of first class, with rank of major of cavalry, for staff, hospital and bureau duties.

Fifty surgeons, second class, with rank of captain of cavalry to be assigned to duty with regiments.

Assistant surgeons, not exceeding seventy, with rank of first lieutenant of cavalry, with duties of assistant surgeons.

Not exceeding seventy-five medical cadets, not less than eighteen nor more than twenty-three years old at their entry, to be examined by a medical board. After three years' continuous service they may be examined for promotion to the rank of the highest class of non-commissioned officers.

As many Hospital Stewards as the service requires, designated by a Sanitary Inspector, on the recommendation of the Senior Surgeon of the post, division, or regiment, with rank of First Sergeants of Cavalry.

Sections 2, 3, 4, 5 and 6, provide for selection by the President, from the whole Army Medical Corps, of suitable persons to fill the places of Director-General, Sanitary Inspector-General, and Sanitary Inspectors, none of whom are to be over sixty years of age. Other officers are to be appointed and promoted by seniority. Vacancies are to be filled from civil life or from Brigade Surgeons of volunteers, after due examination, who are not to be over thirty-five years of age.

Section 5 repeals the allowance of extra rations to Surgeons, upon the completion of ten years' service.

Section 7 provides for the retirement of every medical officer sixty-five years old.

Section 8 repeals all inconsistent laws.

If any one thing is more clear than another it is that some change is requisite in the conduct of medical affairs in the army. Nevertheless it is a dangerous matter to meddle with. If it be tossed into the Congressional bear gardens, who knows but that Mr. Senator Grimes will foist in a homœopathic clause, as he has already threatened a bill to introduce homœopathy into the hospitals at Washington. Many a member, whose seat in House or Senate is "shaky," will seek the smiles and support of some local demagogue at home by prostituting his trust to the support of the demagogue's pet humbug—trusting meanwhile to escape deserved castigation from an insulted profession. *Mark these men*—for the time will come when a good memory may be used to advantage by honest men as well as knaves.

---

*Good Pay for once.*—Dr. Toland, of San Francisco, it is said, enjoys an income of $35,000 *per annum* from the practice of medicine and surgery.

*Chateaux en Espagne.*—The Cincinnati *Lancet* publishes a fee bill in nominal force in that city, which is largely above the rates in this city, remarking however that "these rates are all understood to be simply a maximum rate of fitting remuneration." This leaves the whole matter really at sea, for it is the under-bidding of anxious seekers after practice which demoralizes. Good faith requires that there should be a *minimum* as well as a maximum. As a maximum the Cincinnati rates are much too low, as a minimum it is clear that they cannot be sustained. With our confere, we adopt the language of the Boston *Journal:* "We trust our distant readers will not be deluded into supposing that the average collections of physicians here come anywhere near the aggregate which this table makes their charges assume on their books. Far from it. The grand total recorded may make a very pretty picture, but, alas, it is, in the main, a 'dissolving view,' the 'stuff that dreams are made of," 'the basis for air castles,' *Chateaux en Espagne.*"

---

*Syphilis conveyed by Vaccine Lymph.*—The London *Lancet* records from an Italian journal the transmission of Syphilis to forty-six children by vaccination with contaminated lymph derived from a single source. The evidences of syphilitic infection appear entirely conclusive.

---

*The Excision of Joints.*—By Richard M. Hodges, M. D. Boston, 1861; pp. 204.—This is one of the Boylston Prize Essays, and appears to be a valuable *resume* of the subject. It will be further noticed in an ensuing number.

---

*Dropsical Accumulation.*—Prof. Peaslee has recently removed one hundred and forty-nine pounds and three ounces of dropsical fluid at a single operation. The patient, a young lady, previously had an abdominal circumference of six feet and two inches. Dr. Peaslee in April last removed from the same patient one hundred and thirty-five pounds of fluid.

*Medical Journals.*—So far as we can ascertain there are now but *twelve* medical journals surviving in the United States, and two of them are published on the Pacific coast.

---

*Index of Vol.* 4, 1861.—We suspect that a glance at the list of subjects noticed and discussed in the last volume, the index for which is sent out with this number, will demonstrate that the Medical world still moves, and that active brains are still at work in developing the real power and respectability of the medical profession. It is sincerely believed that, in the year past, the CHICAGO MEDICAL JOURNAL has been both a reflex and index of the progress of the art and science of medicine. No effort will be spared to render the ensuing volume an exponent such as its friends desire and the profession demand. Arrangements are being made by which it is expected that the faults of the mail, which, during the year past, have annoyed both editors and subscribers, will be corrected, and prompt transmission secured.

---

*Mortuary Statistics of Chicago for* 1861.—The subjoined table we reproduce from the *Chicago Daily Times :*

| Months. | Adults. | Male. | Female. | Child'n. | Male. | Female. | Child'n of Nat. Pa's. | Child'n of For. Pa's. | Total |
|---|---|---|---|---|---|---|---|---|---|
| January ......... | 90 | 35 | 55 | 83 | 40 | 43 | 23 | 60 | 173 |
| February ........ | 60 | 36 | 25 | 75 | 36 | 39 | 25 | 50 | 135 |
| March ........... | 85 | 32 | 53 | 87 | 40 | 47 | 28 | 59 | 135 |
| April ........... | 50 | 38 | 12 | 74 | 28 | 48 | 26 | 50 | 126 |
| May ............. | 58 | 28 | 30 | 76 | 50 | 26 | 30 | 46 | 134 |
| June............. | 49 | 30 | 19 | 82 | 39 | 43 | 35 | 47 | 131 |
| July............. | 120 | 65 | 55 | 119 | 85 | 34 | 50 | 69 | 239 |
| August...... .... | 90 | 48 | 47 | 172 | 100 | 72 | 38 | 134 | 262 |
| September ....... | 75 | 40 | 35 | 152 | 85 | 67 | 40 | 112 | 227 |
| October ......... | 55 | 28 | 27 | 65 | 34 | 31 | 22 | 33 | 120 |
| November ........ | 72 | 50 | 22 | 83 | 45 | 38 | 27 | 56 | 155 |
| December........ | 92 | 61 | 31 | 103 | 68 | 35 | 41 | 62 | 195 |
| Totals....... | 896 | 485 | 411 | 1173 | 650 | 523 | 385 | 778 | 2069 |

---

*Mislaid.*—The address of the subscriber who enclosed a ten dollar bill on Grayville Bank. Please write.

